# Traditional living and cultural ways as protective factors against suicide: perceptions of Alaska Native university students

**DOI:** 10.3402/ijch.v72i0.20968

**Published:** 2013-08-05

**Authors:** Christopher R. DeCou, Monica C. Skewes, Ellen D. S. López

**Affiliations:** 1 Department of Psychology, Idaho State University, Pocatello, ID, USA; 2 Department of Psychology, Center for Alaska Native Health Research, University of Alaska Fairbanks, Fairbanks, AK, USA

**Keywords:** Alaska Native, cultural ways, protective factors, suicide, qualitative, interviews

## Abstract

**Introduction:**

Native peoples living in Alaska have one of the highest rates of suicide in the world. This represents a significant health disparity for indigenous populations living in Alaska. This research was part of a larger study that explored qualitatively the perceptions of Alaska Native university students from rural communities regarding suicide. This analysis explored the resilience that arose from participants’ experiences of traditional ways, including subsistence activities. Previous research has indicated the importance of traditional ways in preventing suicide and strengthening communities.

**Method:**

Semi-structured interviews were conducted with 25 university students who had migrated to Fairbanks, Alaska, from rural Alaskan communities. An interview protocol was developed in collaboration with cultural and community advisors. Interviews were audio-recorded and transcribed. Participants were asked specific questions concerning the strengthening of traditional practices towards the prevention of suicide. Transcripts were analysed using the techniques of grounded theory.

**Findings:**

Participants identified several resilience factors against suicide, including traditional practices and subsistence activities, meaningful community involvement and an active lifestyle. Traditional practices and subsistence activities were perceived to create the context for important relationships, promote healthy living to prevent suicide, contrast with current challenges and transmit important cultural values. Participants considered the strengthening of these traditional ways as important in suicide prevention efforts. However, subsistence and traditional practices were viewed as a diminishing aspect of daily living in rural Alaska.

**Conclusions:**

Many college students from rural Alaska have been affected by suicide but are strong enough to cope with such tragic events. Subsistence living and traditional practices were perceived as important social and cultural processes with meaningful lifelong benefits for participants. Future research should continue to explore the ways in which traditional practices can contribute towards suicide prevention, as well as the far-reaching benefits of subsistence living.

Suicide persists as a compelling public health challenge for Alaska Native peoples, and it represents a substantial health disparity for individuals, families and communities in Alaska (1). From 1999 to 2007, the base rate of suicide in the United States was 10.9 deaths per 100,000 people; in contrast, Native peoples in Alaska experienced a rate of 41.3 deaths per 100,000 people (2). Although myriad epidemiological scholarship explicates the magnitude of this disparity (3, 4), there is a lack of research exploring specific protective factors against suicide for people living in rural communities (1). Nevertheless, it has been suggested that such insights are critical to the development of relevant and effective interventions to address the disparate rates of suicide affecting Alaska Native peoples, and those living in rural and indigenous communities (1, 5, 6).

Traditional ways of life in rural Alaska exemplify robust legacies of resilience, creativity and adaptive responsiveness to emerging challenges (7). The resilience described in this article specifically refers to the ability of Alaska Native peoples to engage in traditional ways of life and important cultural practices, and to concordantly pursue progress, achievement and success in Western social, cultural, academic and professional contexts. Previous research highlights the importance of traditional and cultural ways in the development of effective interventions for indigenous communities (8–10). The embrace and integration of cultural practices (such as subsistence hunting and gathering) within contemporary psychological and public health interventions for critical concerns, including suicide, may portend successful outcomes that go beyond conventional models of treatment and intervention (11).

Understanding the strength of traditional ways offers an important path forward for empowering affected stakeholders to address suicidal behavior in tandem with Western research epistemologies. For example, Allen and colleagues (12) described a strengths-based approach to culturally based intervention for suicide and substance abuse, whereby integral systems of traditional living and cultural ways were synthesised to form relevant treatment modalities. These traditional skill sets and cultural knowledge were then applied through collaborative interpretation and application of cultural ways as a metaphor for techniques and strategies that may mitigate problematic behavioral processes, and detrimental sequellae, including suicide (12).

The present study sought to qualitatively explore the thoughts, opinions and experiences of rural university students concerning suicide and suicide prevention in rural Alaska. University students who had migrated from rural Alaska to an urban-university setting offered unique perspectives concerning this difficult and sensitive topic. This article specifically reports findings from the analysis of data related to participants’ perceptions of traditional ways and subsistence activities as protective factors against suicide. Understanding affected stakeholders’ perceptions of this synthesis between traditional practices and contemporary suicide prevention efforts is important to the advancement of on-going research and practice within indigenous cultural contexts.

## Method

### Participants

This study included a purposive sample of 25 college students who self-identified as being “from a rural Alaskan village”. No a priori definition of rurality was prescribed; instead, participants were invited to self-identify as rural based upon their own perceptions of their home communities. Although being Alaska Native was not an inclusion criterion for participation, all participants in the present sample were from Alaska Native ethnic groups. Only students currently enrolled in university classes, and aged 18 years and older were eligible to participate.

The majority of participants were female (n=18), ranging in age from 18 to 37 years (M=23.64; SD=4.61). Participants included students who still considered their home village to be their permanent residence when not attending classes at the university, as well as students who had relocated from their home village 21 years prior to completing an interview (M=4.68; SD=5.24). All students self-identified as being Alaska Native and from rural villages with populations, ranging from 70 to 6,500. Almost all participants (n=23) reported being from villages where another language was spoken in addition to English. However, all participants were themselves university students who spoke English fluently.

### Procedure

This study used in-person individual interviews to qualitatively explore participants’ thoughts, opinions and experiences related to the problem of suicide in rural Alaska. Due to the far-reaching impact of suicide in rural Alaska and ethical concerns related to asking participants about their personal experiences related to suicide, the study included a safety plan to address participant distress and provided a list of crisis counselling resources to all participants. However, it is important to note that there were no adverse reactions among participants in the interview study; the safety plan was not needed, and participants reported their experiences to be meaningful and beneficial, despite being difficult (13). Students were offered $50 as compensation for their time and effort. After obtaining informed consent, all participants completed a brief demographics and background questionnaire. The university's Institutional Review Board approved all methods and materials.

The first author conducted most of the interviews (n=19), except for those participants (n=6) who requested a female interviewer. The first author had prior training and experience in conducting interviews with survivors of trauma and provided training for the female interviewer prior to her first interview. Preparation for conducting interviews included discussion of interviewer style and setting, rehearsal interviews, discussion of the safety plan and challenges that may arise during an interview.

### Interview protocol

The interviews were conducted using a semi-structured interview guide, developed specifically for this study, in collaboration with cultural and community advisors connected with the university's rural student community. The interview questions represented a progression through 5 conceptual domains, including background (e.g. What is the best part of life in the community you are from?), rural–to–urban transition (e.g. What steps did you have to take to attend college at [University]), suicide in rural Alaska (e.g. In what ways has suicide affected your life?), moving forward (e.g. What is your dream or vision for the future of the community you are from?) and debriefing (e.g. Compared to when you arrived, do you feel better, the same, or worse?). This progression allowed the interviewer to establish rapport, understand context, discuss experiences related to suicide and conclude the interview experience with a strengths-based discussion of the participants’ personal journeys to college. The interviews included open-ended questions, using a non-judgmental and empathic listening style. Interviews lasted an average of 1 hour, and were audio-recorded with participant consent.

The interview protocol included specific questions concerning the importance of culture and traditional ways. Given the findings of previous research regarding the centrality and protective value of traditional and cultural ways among Alaska Native Peoples (14), interview questions were phrased to highlight the strength that may arise from buttressing cultural practices. These questions consisted of (a) “In what ways do you feel most connected to the traditions and heritage of your home village and elders?”; (b) “What cultural traditions can be strengthened to prevent suicide?”; (c) “How are people able to live healthy lives in the village you're from?”; and (d) “If somebody is depressed in your home community, what could they do to get better?”

### Recruitment

Participants were recruited through the use of flyers posted on campus, including student dormitories, academic buildings and the Office of Rural Student Services. Flyers were also distributed through student organisations that specifically serve rural and indigenous students. Posted flyers provided students with basic details about the scope of the study, including the topic of suicide and compensation for participation. Upon contacting the first author, participants were screened for eligibility and offered the choice of scheduling an interview with a male or female interviewer.

Unintentionally, snowball sampling also occurred. That is, once data collection began, several participants mentioned being referred by peers who had already completed an interview. Some participants explicitly stated that their intention to complete an interview was to determine whether or not they would recommend the interview experience to other rural students. All reported that they would be referring other rural students.

### Analysis

Members of the research team transcribed the interviews verbatim. Transcripts were de-identified to protect the confidentiality of participants, home villages and others mentioned during the interviews. The interviews were analysed using the techniques of grounded theory (i.e. open-coding and constant comparison) for the purpose of identifying salient themes and concepts within and across participant transcripts (15, 16).

## Findings

Several key themes emerged from the analysis of transcripts, and they revealed participants’ appreciation of traditional ways and subsistence activities as integral to the unique quality of life in rural communities, as well as central to efforts to prevent suicide. Traditional ways were thought to facilitate important relationships, promote healthy living, contrast with contemporary challenges and communicate important cultural values.

### Context of important relationships

Participants expressed the importance of family and mentor relationships in the process of mitigating suicide risk and addressing social isolation. Traditional ways and subsistence activities represented an important avenue for those relationships and appeared to position key relationships within community, familial and dyadic contexts. That is, traditional ways integrated critical social processes within tight knit villages across multiple ecological levels. As 1 participant explained concerning relationships among community members:

The best part of life, I grew up there, and I liked it very much, pretty much livin’ the cultural ways, bein’ able to still do those cultural things, like subsistence living, Native dancing, and stuff like that. And they have celebrations every other fall for families who have family members that are deceased, and they throw potlatches, and that's a way to get the community together and to get them sharing, and just being involved, and stuff like that. (P08)

In addition to contextualising relationships among community members, traditional hunting and gathering activities represented a central experience within families for many participants. One participant explained the trans-generational importance of annual subsistence activities for their family:

I was pretty much raised by my parents and my grandparents, my maternal grandparents, we would go camping every August for a week, pick berries, and while we're picking berries the men would trap, we'd eat fish and drink tundra tea, and just living, the way our ancestors did. (P06)

Another participant explained the instructive role of their mother in supporting connection with elders, who represent important mediators of cultural knowledge and traditions: “My mom used to make me visit Elders in [home village], I'm glad she did, um, and they were cool folks, the ones I used to visit.” (P05)

### Promotes healthy living to prevent suicide

In addition to the meaningful relationships integral to traditional ways and subsistence activities, participants also identified the ways in which these cultural practices promote healthy living. In this way traditional practices and subsistence activities represented a means by which the antecedents of suicidal behavior (e.g. depression) might be attenuated. One participant described access to Native foods as a way to pursue health within the context of a village community: “I guess healthy eating, we have fish, we have a lot of fish. Like I said, there is some subsistence fishing, people go hunting so there's fresh meat. I guess that's one aspect of healthy living” (P07). In addition to traditional foods, participants also explained the importance of interaction with nature, and experiences involving tribal and village lands. One participant described a nature walk as a way to be healthy within a rural context:

I would suggest more nature walks … Just walking up the hill, like all the way up. It gets really windy, but then like it takes like an hour to walk all the way up, maybe hour and a half, and take a camera, take pictures, pick flowers, I think that's how they could be more healthy. (P06)

It is important to note that these recommendations for healthy living occurred within the context of participants’ responses to interview questions specific to the problem of suicide in rural Alaska.

### Contrast with current challenges

Although participants described the importance of cultural ways, they also expressed their perceptions of a schism between traditional ways and modern lifestyles in rural communities. One participant shared their observation concerning these changing paradigms:

I think that a healthy lifestyle would be like back in the old days, where there was no alcohol, no drugs, no disrespect, and livin’ off only the land, and think that would be like the healthiest lifestyle to live in the villages … because people back then would respect everything and everyone more than now. (P10)

In addition to perceived changes in the relationships between people and natural resources, participants also described their observations concerning changes in family relationships. This participant observed a decrease in the relationships between children and grandparents, and concordantly identified diminished participation by children in subsistence activities:

There was that tie between the young children and the grandparents that I don't see too much anymore. You see it in select families and those are the families you feel like are the more healthy ones. But, it seems like now, all the young generation kids, they don't want to spend time doing anything subsistence. (P03)

### Communicate important cultural values

In addition to facilitating the contexts of relationships, participants described traditional ways and subsistence activities as cultural metaphors necessary for passing cultural knowledge from one generation to the next, particularly in rural communities. One participant described the multiple layers of practical importance and transmission of cultural values associated with subsistence activities:

It's a way of life that you can pass down to your children, in a way that you can't in the city or in a small town … not only are you passing down how to hunt and fish but you're also teaching different family morals as well. (P01)

Participants explored cultural values in nuanced ways that recognised the changing realities of rural Alaska, and concurrently highlighted the need to support and communicate values in a sustainable way. One participant described their idea to instil and support traditional cultural values, including the integration of subsistence:

I had the idea of bringing back the [tribal] values, like from back when people used to rely on each other and respect each other. There's the language and there's subsistence and I don't think we're gonna bring back the spirit world but we can bring back respect. (P10)

Although many participants described the decline of traditional values within rural communities, they remained hopeful about the potential for reclaiming and re-appropriating these cultural ways to address health concerns, such as suicide. One participant described the potential for instilling the value interdependence among youth through the use of traditional dance and languages:

I think if they, if we, tried to strengthen the language and our Native dance it would bring the community more together, and um, the youth would be involved a lot, and they'd know from a younger age that they're a part of something, and, um, they'd have people to lean on if they needed. (P18)

## Discussion

Taken together, participants’ perceptions of traditional ways and subsistence activities represent a synthesis of relationships, health and culture. Participants viewed traditional ways as meaningful and beneficial aspects of rural living with potential applications to intervention and prevention strategies for individuals, families and communities. The findings of this article are commensurate with previous research conducted with Alaska Native and indigenous peoples (8, 9, 12, 17) and offer additional insight into the perceptions of affected stakeholders. Specifically, these findings explicate participants’ perceptions of traditional and cultural ways as protective against suicide, and support the imperativeness of additional research to evaluate the benefits of framing suicide prevention efforts within the strengths and resilience of Alaska Native Peoples. However, it is important to consider the limitations of the present findings.

### Limitations

The findings presented in this article emerged from interviews with Alaska Native students who self-selected to participate in this study. It is possible that these participants are not representative of other rural and indigenous students in Alaska, and they may be substantially different from those students choosing not to participate in a study they knew would discuss the topic of suicide. Moreover, the selection bias that limits these findings may be more pronounced given the unintentional snowball sampling that occurred. It is also important to consider the potential limitation that may arise from underestimating heterogeneity within the specific cultural groups and regions represented within our sample of Alaska Native university students. Indeed, our participants represented a diversity of rural and indigenous communities across the State of Alaska.

## Conclusion

Traditional ways and subsistence activities were perceived as important aspects of participants’ experiences, and considered protective against suicide. Participants described the changing nature of rural communities, and possible challenges to the sustainability of these cultural practices. However, participants remained hopeful that concerted efforts could promote the continued integration of traditional ways and subsistence activities within contemporary efforts to address the problem of suicide in rural Alaska. Additional research should include quantitative and prospective methods to determine the extent to which the inclusion of cultural and traditional ways in treatment and prevention may yield advantageous outcomes beyond Western models of health and healthcare alone.
